# Bta-miR-365-3p-targeted FK506-binding protein 5 participates in the AMPK/mTOR signaling pathway in the regulation of preadipocyte differentiation in cattle

**DOI:** 10.5713/ab.23.0328

**Published:** 2024-04-24

**Authors:** Mengdi Chen, Congcong Zhang, Zewen Wu, Siwei Guo, Wenfa Lv, Jixuan Song, Beibei Hao, Jinhui Bai, Xinxin Zhang, Hongyan Xu, Guangjun Xia

**Affiliations:** 1 College of Agriculture, Yanbian University, Yanji 133000, China; 2 College of Integration Science, Yanbian University, Yanji 133000, China; 3 Institute of Animal Science, Jilin Academy of Agricultural Science, Changchun 136100, China; 4 Joint Laboratory of Modern Agricultural Technology International Cooperation, Ministry of Education, Jilin Agricultural University, Changchun 130118, China; 5 Research Center of North-East Cold Region Beef Cattle Science & Technology Innovation, Yanbian University, Yanji, Jilin 133000, China

**Keywords:** AMPK/mTOR Signalling Pathway, bta-miR-365-3p, FK506-binding protein 5 (*FKBP5*), Preadipocytes, Yanbian Yellow Cattle

## Abstract

**Objective:**

MicroRNAs (miRNAs) are endogenous non-coding RNAs that can play a role in the post-transcriptional regulation of mammalian preadipocyte differentiation. However, the precise functional mechanism of its regulation of fat metabolism is not fully understood.

**Methods:**

We identified bta-miR-365-3p, which specifically targets the 3′ untranslated region (3′UTR) of the FK506-binding protein 5 (FKBP5), and verified its mechanisms for regulating expression and involvement in adipogenesis.

**Results:**

In this study, we found that the overexpression of bta-miR-365-3p significantly decreased the lipid accumulation and triglyceride content in the adipocytes. Compared to inhibiting bta-miR-36 5-3p group, overexpression of bta-miR-365-3p can inhibit the expression of adipocyte differentiation-related genes *C/EBPα* and *PPARγ*. The dual-luciferase reporter system further validated the targeting relationship between bta-miR-365-3p and FKBP5. FKBP5 mRNA and protein expression were detected by quantitative real-time polymerase chain reaction and Western blot. Overexpression of bta-miR-365-3p significantly down-regulated FKBP5 expression, while inhibition of bta-miR-365-3p showed the opposite, indicating that bta-miR-365-3p negatively regulates FKBP5. Adenosine 5′-monophosphate (AMP)-activated protein kinase/mammalian target of rapamycin (AMPK/mTOR) signaling pathway is closely related to the regulation of cell growth and is involved in the development of bovine adipocytes. In this study, overexpression of bta-miR-365-3p significantly inhibited mRNA and protein expression of *AMPK*, *mTOR*, and *SREBP1* genes, while the inhibition of bta-miR-365-3p expression was contrary to these results. Overexpression of FKBP5 significantly upregulated *AMPK*, *mTOR*, and *SREBP1* gene expression, while inhibition of FKBP5 expression was contrary to the above experimental results.

**Conclusion:**

In conclusion, these results indicate that bta-miR-365-3p may be involved in the AMPK/mTOR signaling pathway in regulating Yanbian yellow cattle preadipocytes differentiation by targeting the *FKBP5* gene.

## INTRODUCTION

In beef cattle production, adipose tissue deposition directly affects its meat production performance, meat quality and nutritional value. Increasing the intramuscular fat deposition can effectively improve meat quality traits [1]. Intramuscular fat content affects the flavor, tenderness, color, and juiciness [2]. In turn, it is influenced by a number of factors, including genetic factors (e.g., breed, sex, and genotype) and non-genetic factors (e.g., castration, weight, age, environment, management, and nutrition) [3]. Yanbian yellow cattle mainly live in Yanbian Korean Autonomous Prefecture, Jilin Province, China, which are strong, hardy, adaptable and have stable genetic traits. They can produce tender, tasty beef that is favored by consumers in local and other regions [4].

Adipose deposition is caused by an increase in the number and size of adipocytes, derived from mesenchymal stem cells (MSCs). MSCs first differentiate into adipoblasts, then into preadipocytes, and finally into mature adipocytes [5]. The differentiation process is regulated by a variety of transcription factors. During the early stages of adipogenesis, lipogenic transcription factor CCAAT enhancer-binding proteins (C/EBPs), peroxisome proliferator-activated receptors (PPARs) and sterol-regulatory element binding proteins (SREBPs) play important roles [6], C/EBPβ and C/EBPδ stimulate the expression of PPARγ and C/EBPα [7], enabling PPARγ and C/EBPα to participate in the final differentiation process of adipocytes [8].

microRNAs (miRNAs), a class of non-coding single-stranded RNA molecules encoded by endogenous genes of about 22 to 25 nucleotides long, have been shown to act as post-transcriptional regulators of gene expression [9]. Most miRNA genes are present in the genome as single-copy, multi-copy, or gene clusters that function by pairing with the 3′ untranslated region (3′UTR) of the target mRNAs, inhibiting translation, or targeting the mRNA for degradation [10]. Many studies have confirmed that miRNAs are involved in the molecular regulation of adipose tissue development and lipid metabolism. The first miRNA described to be associated with adipogenesis was miR-143, which regulated adipogenes via extracellular signal-regulated kinase 5 (ERK5) [11]. As research progressed, more and more miRNAs were found to be involved in adipogenesis. Xu et al [12] found that miR-381 promoted the differentiation of bovine preadipocytes by targeting the expression of KCTD15. Ma et al [13] showed that bta-miR-130a and bta-miR-130b reduced lipid droplet accumulation and inhibited bovine adipocyte differentiation by targeting the 3′UTR of PPARγ and CYP2U1. Chen et al [14] found that the miR-540 directly targeted the 3′UTR of the PPARγ, resulting in a decreased expression of the PPARγ protein and inhibition of adipocyte differentiation.

The research group previously conducted a transcriptome analysis of miRNA-seq and RNA-seq in longissimus dorsi muscle of 36-month-old yanbian yellow cattle and steers and found that bta-miR-365-3p was differentially expressed between them, among which the steers had higher intramuscular fat than the cattle [15]. The FK506-binding protein 5 (*FKBP5*) gene was predicted by Targetscan as a target of bta-miR-365-3p. In previous studies, miR-365-3p was shown to be expressed at a high level in muscle tissues [16]. In Japanese black cattle, bta-miR-365-3p was differentially expressed between semitendinosus and occluder muscles [17]; and in the proliferative stage of skeletal muscle-derived satellite cells of Chinese Simmental calves, the number of sequence reads of bta-miR-365-3p was 3.5 times [18]. To our knowledge, no reports link bta-miR-365-3p and any of its target genes to adipogenesis, therefore, we speculated that bta-miR-365-3p and FKBP5 might affect intramuscular fat deposition in Yanbian yellow cattle.

In this study, we used preadipocytes of 7-day-old Yanbian yellow cattle to study adipogenesis. Quantitative real-time polymerase chain reaction and western blot techniques were used to detect the expression of PPARγ, C/EBPα, and FKBP5. Lipid accumulation was detected using oil-red O staining, and a dual-luciferase reporter assay was performed to identify and validate potential interactions associated with the targeting relationship between bta-miR-365-3p and FKBP5. This study aimed to elucidate the role of bta-miR-365-3p in adipocyte differentiation, and the related target genes were obtained and the ways of its participation in regulating intramuscular fat deposition were explored, which provided a theoretical basis for producing high-quality beef.

## MATERIALS AND METHODS

### Specimen collection and isolation of bovine preadipocytes

All experimental procedures for this experiment were carried out in accordance with the guidelines formulated in the Regulations on the Management of Experimental Animals (Ministry of Science and Technology, China, 2017) and approved by the Medical Ethics Committee of the School of Medicine of Yanbian University (Approval No.: 2375). The preadipocytes of Yanbian yellow cattle selected in this experiment were provided by the College of Agriculture, Yanbian University (Yanji, Jilin, China). The cattle used for the experiment were one week old Yanbian yellow cattle. Preadipocytes were taken from its axilla.

Primary preadipocytes of Yanbian yellow cattle were isolated by two-step enzyme digestion. Fibers and blood vessels in adipose tissue were cut on a sterile ultra-clean table, soaked with 75% alcohol for 10 min, and washed three times with phosphate-buffered saline (PBS) (Biological Industries, Beth HaEmek, Israel) containing 2% penicillin-streptomyc in mixture. Adipose tissue was cut into 1 mm^3^ and weighed. Type IV collagenase at a concentration of 0.1% of its 2-fold volume was added per gram of tissue. After the centrifuge tube was inverted to ensure that the tissue was fully infiltrated by the digestive juice, the tissue was gently digested in a water bath at 37°C for 60 min and mixed reversely once every 5 min. After digestion, Dulbecco’s modified eagle medium (DMEM) (Gibco, Sacramento, CA, USA) growth medium (10% fetal bovine serum [FBS], 89% DMEM, 1% penicillin-streptomycin mixture) in the same volume as the digestion solution was added to terminate the digestion, centrifuge at 1,500 r/min for 10 min and discard the supernatant; the precipitate was resuspended with growth medium and then passed through a sterile filter of 100 and 200 mesh pore size in turn, centrifuge the filtrate at 1,500 r/min for 10 min and discard the supernatant. The precipitate was resuspended with growth medium and then inoculated into 25 cm^2^ culture flasks at 37°C and 5% CO_2_ saturation humidity.

### Cell culture and transfection

When the adipocyte density was up to 80% to 90%, they were digested with trypsin and then inoculated into sterile six-well plates at the appropriate density, and cultured with DMEM (Gibco, USA) growth medium at 37°C with 5% CO_2_. Cells were grown to 60% and cultured with Opti-MEM medium (Gibco, USA) for 4 h without antibiotics, differentiated when the cell density reached about 80%. Preadipocytes were then transfected with bta-miR-365-3p mimics, negative control (Ribobio, Guangzhou, China) at a concentration of 50 nM, bta-miR-365-3p inhibitors (Ribobio, China) were cultured using 5 μL lipofectamine 2000 (Invitrogen, Carlsbad, CA, USA).

### FKBP5-siRNA sequence, overexpression vector design and inhibition efficiency assay

According to the mRNA sequence of the *FKBP5* gene queried in GenBank, three siRNA targets 308,453, and 819 were selected for the FKBP5 inhibitory expression design, respectively named FKBP5-Bos-308, FKBP5-Bos-453, and FKBP5-Bos-819. A nonsense codon sequence was used as a control and synthesized by Shanghai Gemma Company (Shanghai, China), and Table 1 is the primer sequence.

The FKBP5 overexpression vector (Figure 4E) was synthesized by Shanghai Gemma Company (Shanghai, China) based on the CDS region of FKBP5 mRNA sequence. The plasmid vector used in the overexpression vector is pcDNA3.1(+), and the restriction sites are BamHI and EcoRI.

When preadipocytes grew to 80% to 90%, they were digested with trypsin and then inoculated into a sterile 6-well plate. The six-well plate was incubated in a temperature incubator at 37°C and saturated humidity at 5% CO_2_. When cells grew to 60%, the medium was replaced with duplex antibody containing 10% FBS (Biological Industries, Israel) and siRNA transfection was initiated. Two sterile Ep tubes were prepared inside the console and 250 μL Opti-MEM (Gibco, USA) was added to each tube. One tube was added with 5 μL lipofectamine 2000 (Invitrogen, USA) transfection reagent and left to stand for 5 min, another tube was added with siRNA or negative control, mixed, and left to stand for 20 min. The liquid in the two tubes was mixed and evenly added to a six-well plate, incubated for 24 h and then RNA was extracted, the inhibition effect was measured by quantitative real-time polymerase chain reaction (qRT-PCR).

### Induction of cell differentiation and oil red O staining

Preadipocytes were grown to 80% to 90% and seeded into 6-well plates, cultured in 37°C, 5% CO_2_ of DMEM (Gibco, USA) growth medium, and replaced every 2 days. When cells were fused, the growth medium was replaced with an equal volume containing 0.5 mmol/L-3-xanthine (Sigma-Aldrich, St. Louis, MO, USA), 1 mol/L dexamethasone (Sigma-Aldrich, USA), 10 g/mL insulin (Sigma-Aldrich, USA) in the induction differentiation medium I. After 2 days, the induction differentiation medium I was replaced with induction differentiation medium II (10 g/mL insulin) until the cells were fully differentiated. Cells were washed twice with PBS (Biological Industries, Israel) and fixed with 4% paraformaldehyde for 30 min at room temperature. After fixation, the cells were washed twice with PBS (Biological Industries, Israel) and stained with oil red O (Sigma-Aldrich, USA) working solution for 30 min at room temperature, then the excess staining solution was removed, and the cells were observed by microscope.

### Triglyceride determination

Yanbian yellow cattle preadipocytes were seeded into six-well plates, the cells induced to differentiate until day 9 were washed with PBS (Biological Industries, Israel) and 800 μL-1 mL PBS (Biological Industries, Israel) was added to each well. The cells were scraped down with a cell scraper and transferred to a sterile 1.5 mL centrifuge tube. Then they were centrifuged at 1,000 r/min for 10 min, and bta-miR-365-3p mimics, inhibitor, and negative control (Ribobio, China) transfected cells were collected. Added 200 μL, 2% TritonX-10 0 lysate to each tube and lysed for 35 min. Triglyceride content was determined according to a commercial kit (Blue Sky, Shanghai, China).

### RNA extraction and quantitative real-time polymerase chain reaction

RNA were extracted from the preadipocytes using the Eastep Super Total RNA extraction kit (Promega, Shanghai, China) and reverse transcribed to cDNA with the FastKing gDNA Dispelling RT SuperMix kit (TIANGEN, Beijing, China). The qRT-PCR was performed using the SuperReal PreMix Plus kit (TIANGEN, China), and the qRT-PCR reaction solution was prepared on ice: 2 SuperReal PreMix Plus 20 μL, 1.2 μL for each of the positive and negative primers, 4 μL for the cDNA template, 50 ROX Reference Dye 0.8 μL, and RNase-free ddH_2_O 12.8 μL. The qPCR reaction was performed in a PCRmax Eco 48 real-time PCR instrument (PCRmax, Stone, UK), with the reaction procedure: pre-denaturation at 95°C for 15 min, denaturation at 95°C for 10 s and annealing at 60°C for 30 s for a total of 40 cycles. All the experiments were performed three times independently. The 2^−ΔΔCt^ method was used to calculate relative mRNA expression and β-actin as a housekeeping gene to regulate the expression of other protein-coding genes. The primer sequences used for qRT-PCR are shown in Table 2.

### Protein extraction and Western blot analysis

Preadipocytes were incubated with RIPA Lysis Buffer (Beyotime, Beijing, China) containing 1 mM phenylmethanesulfonyl fluoride (Beyotime, China) on ice. Total protein concentration of the cell lysates was determined by Enhanced BCA protein Assay Kit (Beyotime, China) according to the manufacturer’s instructions. Protein samples were electrophoresed using 10% sodium dodecyl sulfate-polyacrylamide gel electrophoresis gels (Solarbio, Beijing China) with 20 μg of protein per well, a polyvinylidene fluoride (PVDF) membrane (Millipore, Billerica, MA, USA) of the same area as the gel was cut. The PVDF membrane was activated in methanol for 30 s and then put into the membrane transfer solution, and the membrane was transferred at constant pressure and 0.2A. After completion, the PVDF membrane was rinsed with TBS containing 0.1% Tween 20 (TBST) and blocked with TBST containing 5% ovalbumin (Solarbio, China) on a shaker at 22°C±3°C for 2 hours. The PVDF membrane was then incubated overnight with the primary antibody (Supplementary Table S1) at 4°C in blocking buffer and washed five times with TBST. And then incubated with the horseradish peroxidase-conjugated secondary antibody for 2 h at 4°C. The PVDF membrane was washed with TBST, and the immunoblots were prepared using the chemiluminescent solutions, and analyzed using the Alliance MINI HD9 AUTO Western Blot Imaging System (UVITEC, Cambridge, UK). The intensities of the target protein bands were normalized as the intensity of the-actin bands and calculated via the ImageJ program. All experiments were conducted in 3 replicates.

### Luciferase reporter assay

TargetScan (https://www.targetscan.org/vert_80/) and miRBase (https://www.mirbase.org/) were used to predict the target gene of bta-miR-365-3p. According to the prediction, FKBP5 is a potential target gene of bta-miR-365-3p. The wild-type 3′UTR of FKBP5 containing bta-miR-365-3p binding site and the mutant sequence of the binding site were cloned into Psicheck2 vector (Hunan Fenghuishengwu Technology Company, Hunan, China). We call them FKBP5-wt and FKBP5- mut respectively. The vector was transfected into HepG2 cells using Lipofectamine 2000 (Invitrogen, USA) along with a bta-miR-365-3p mimic or NC, the six experimental groups are as follows:

i) Psicheck2 and miR-365-3p mimics
ii) Psicheck2 and miR-NC
iii) Psicheck2-FKBP5-WT and miR-365-3p mimics
iv) Psicheck2-FKBP5-WT and miR-NC
v) Psicheck2-FKBP5-MUT and miR-365-3p mimics
vi) Psicheck2-FKBP5-MUT and miR-NC

At 48 h post-transfection, luciferase activity was detected using the Dual-Glo Luciferase Assay System (Promega, China) and quantified using a luminometer (GloMax 20/20; Promega, China).

### Statistical analysis

All results were expressed as mean±standard error of the mean, and statistical analysis was performed using SPSS 26.0 software. Differences between experimental and control groups were determined by one-way analysis of variance test, and Duncan’s multiple range test for multiple comparisons, * p<0.05, ** p<0.01 as statistically significant.

## RESULTS

### bta-miR-365-3p is highly conserved in mammals

The bta-miR-365 encodes and produces two mature miRNA, bta-miR-365-5p and bta-miR-365-3p, located in cattle on chromosomes 25 and 19 respectively. The bta-miR-365-3p, as one of the maturity bodies of bta-miR-365, was homologous between hsa-miR-365-3p, ssc-miR-365-3p, cgr-miR-365-3p, mmu-miR-365-3p, mml-miR-365-3p, and rno-miR-365-3p.

**Figure 1:**
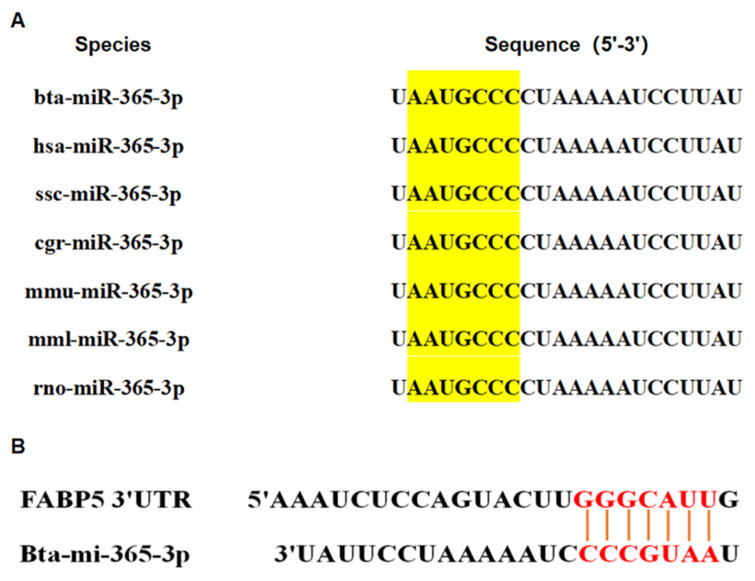
Homologous sequence of miR-365-3p target gene and bioinformatics prediction. (A) miR-365-3p sequence in mammals, and the yellow region is the seed region. Bta, *Bos Taurus*; has, Homo Sapiens; ssc, *Sus Scrofa*; cgr, Cricetulus Griseus; mmu, Mus Musculus; mml, Macaca Mulatta; rno, Rattus Norvegicus. (B) The target site for bta-miR-365-3p in the 3′-UTR of the FKBP5 mRNA (red).

After comparing the miRNA sequences of human, wild boar, mouse, macaque, and other animals, it was found that the mature bta-miR-365-3p sequence was highly conserved among many mammals. The mature miR-365-3p sequence showed no differences in mammals, the seed sequences were identical as well (Figure 1A). The bta-miR-365-3p target gene predicted by TargetScan was compared with the previously described differentially expressed genes determined by RNA-seq analysis. In this process, FKBP5 was identified as a candidate target. The predicted bta-miR-365-3p targets in FKBP5 mRNA sequence (Figure 1B).

### bta-miR-365-3p inhibited preadipocyte differentiation in Yanbian yellow cattle

**Figure 2:**
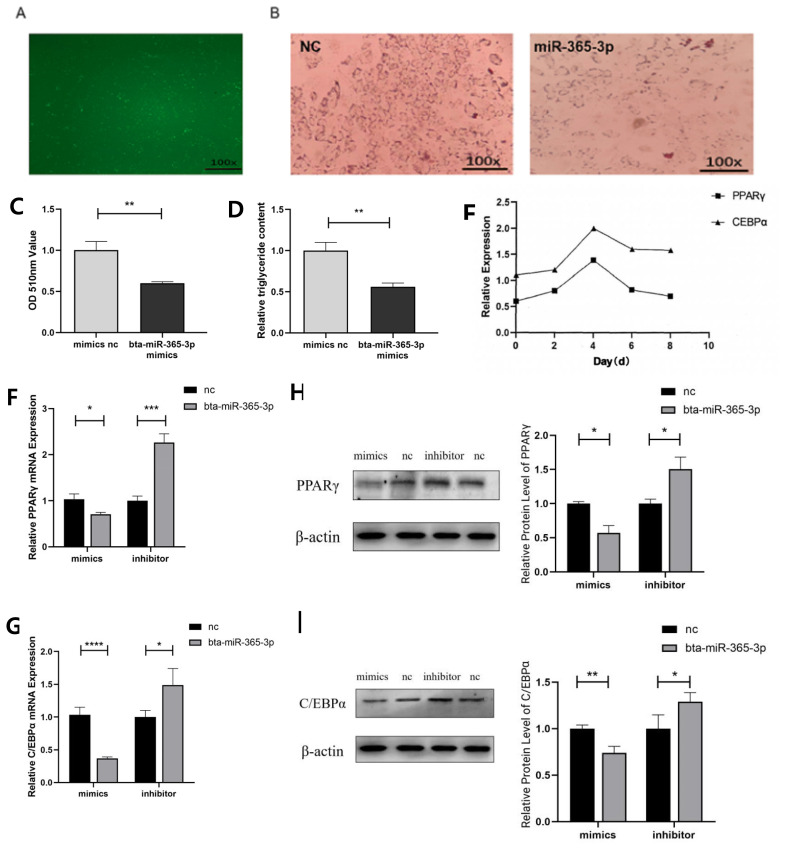
bta-miR-365-3p inhibited preadipocyte differentiation in Yanbian yellow cattle. (A) Preadipocytes were transiently transfected with FAM-labeled bta-miR-365-3p mimics, and transfection efficiency was estimated at baseline by fluorescence microscopy at 4 h later. (B) Adipocytes were stained with oil red O. (C) The lipid content of differentiated cells was quantified by extracting oil red O with isopropanol and measuring its absorption at 510 nm. (D) Cellular triglyceride content was analyzed by a microplate reader. (E) Changes in PPARγ and C/EBPα mRNA levels during preadipocyte differentiation. (F, G) mRNA expression levels of adipogenic markers (PPARγ and C/EBPα). (H, I) The protein level of PPARγ and C/EBPα in adipocytes transfected with bta-miR-365-3p mimics, inhibitors and negative control. * p<0.05; ** p<0.01; ***, **** p<0.001.

After 4 hours, the transfection efficiency of FAM-labeled miRNA was observed under fluorescence microscope, and the transfection efficiency was high (Figure 2A). On the 9th day of differentiation, the preadipocytes of the experimental group and the control group were stained with oil red O and compared by microscope (Figure 2B). The formation of lipid droplet transfected with bta-miR-365-3p mimics significantly reduced (p<0.05) (Figure 2C), and the triglyceride content was significantly less than control group (p<0.05) (Figure 2D). All the results showed that overexpression of bta-miR-365-3p could significantly inhibit lipid accumulation during preadipocyte differentiation in Yanbian yellow cattle. Meanwhile, to explore the expression trend of lipid metabolism marker genes in Yanbian yellow cattle preadipocytes during differentiation, the expression of PPARγ and C/EBPα mRNA was detected by qRT-PCR on the 0, 2, 4, 6, and 8 days of differentiation. The results showed that the expression of PPARγ and C/EBPα mRNA reached the highest value on the fourth day of induced differentiation (Figure 2E). Therefore, on the 4th day of induced differentiation, we detected the contents of PPARγ and C/EBPα mRNA and the corresponding proteins in preadipocytes of different treatment groups by qRT-PCR and Western blotting. The results showed that compared with the control group, the mRNA in the overexpression experimental group decreased significantly (p<0.05), while the expression in the inhibition experimental group had the opposite result (Figure 2F, 2G). At the same time, compared with the control group, the expression of protein in the overexpression experiment decreased significantly (p<0.05) (Figure 2H, 2I). To sum up, these experiments further verified that bta-miR-365-3p can inhibit adipogenic differentiation of Yanbian yellow cattle preadipocytes.

### bta-miR-365-3p directly targets the 3′UTR of FKBP5 mRNA

**Figure 3:**
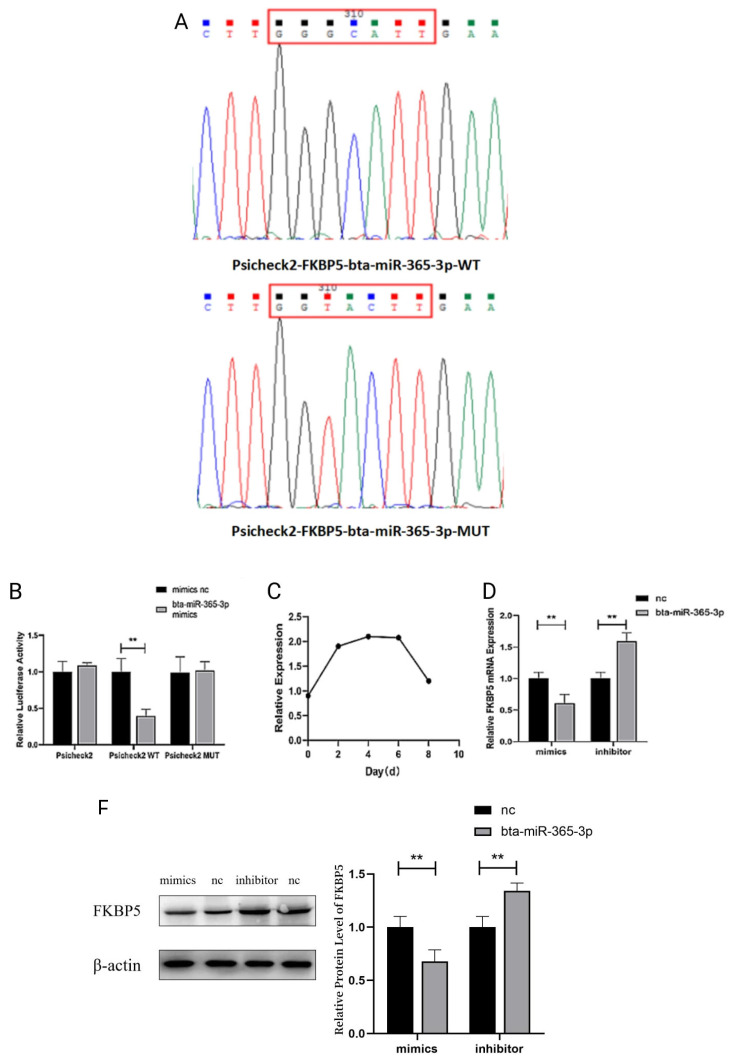
bta-miR-365-3p targets the 3′ UTR of FKBP5. (A) The bta-miR-365-3p-binding region in the FKBP5 3′ UTR that was cloned into Psicheck2-Control to create Psicheck2-Control-WT. The three nucleotides in the bta-miR-365-3p-binding region were mutated to create Psicheck2-Control-MUT. (B) Luciferase activity in HepG2 cells transfected with Psicheck2-Control and pRL-TK dual luciferase-reporter vectors. (C) Changes in mRNA expression of *FKBP5* gene increased with days of induced differentiation. (D–E) Expression of FKBP5 mRNA and protein for preadipocytes transfected with bta-miR-365-3p mimics, inhibitors, and negative control. * p<0.05; ** p<0.01.

To determine whether bta-miR-365-3p directly targets FKBP5 mRNA, Psicheck2-FKBP5-miR-365-3p-wild type (WT) and Psicheck2-FKBP5-miR-365-3p-mutant type (MUT) vectors were created (Figure 3A).

After transfection, HepG2 cells transfected with bta-miR-365-3p mimics showed a significant decrease (p<0.05) in the dual luciferase fluorescence activity of the wild-type vector plasmids, which did not inhibit the mutant and null-loaded plasmids. The effects on all plasmids were not significant (p>0.05) in the control group transfected with mimics NC (Figure 3B). This result suggests a targeting relationship between bta-miR-365-3p and *FKBP5* gene.

In addition, FKBP5 expression showed an increasing trend during preadipocyte differentiation, with peak mRNA expression at day 4 (Figure 3C). In preadipocytes, FKBP5 mRNA and protein expression were detected by qRT-PCR and Western blot, and both mRNA and protein expression were significantly lower in the overexpression experimental group compared with the control group (p<0.05), while the opposite result was observed in the inhibited expression experimental group (Figure 3D, 3E). These data demonstrate that FKBP5 is a direct target of bta-miR-365-3p.

### *FKBP5* gene promotes preadipocyte differentiation in Yanbian yellow cattle

**Figure 4:**
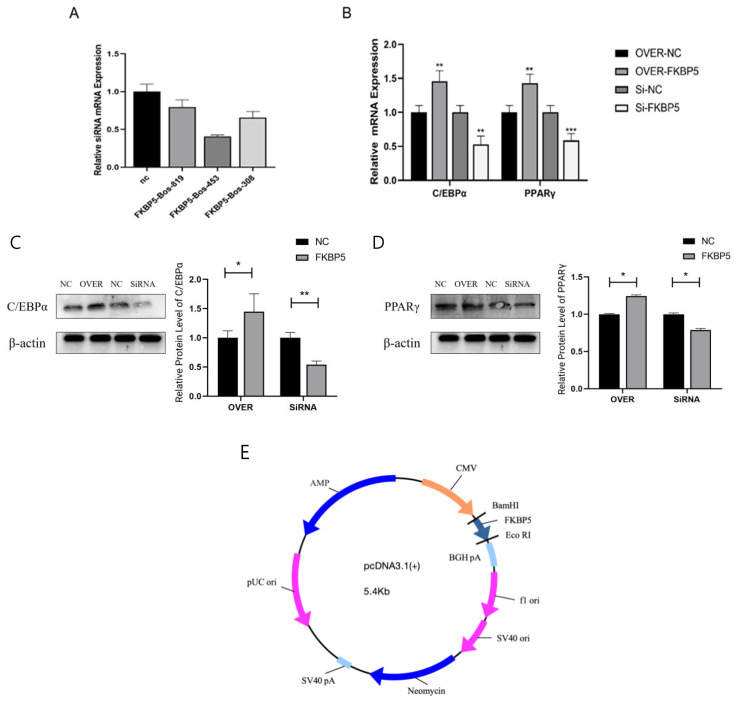
*FKBP5* gene promotes preadipocyte differentiation in Yanbian yellow cattle. (A) Schematic representation of the comparative siRNA transfection efficiency of FKBP5. (B) Effects of overxpression and inhibition of FKBP5 expression on *C/EBPα* gene and *PPARγ* gene mRNA expression in preadipocytes. (C, D) Effects of overexpression and inhibition of FKBP5 expression on *C/EBPα* gene and *PPARγ* gene protein expression in preadipocytes. (E) Overexpression vector of FKBP5. * p<0.05; ** p<0.01.

The results showed that FKBP5-Bos-453 had the best inhibitory effect (Figure 4A), and FKBP5-Bos-453 was selected to inhibit FKBP5 expression in subsequent experiments.

RNA and protein were extracted on the fourth day of induction differentiation, and the changes of C/EBPα and PPARγ expression were detected. The results showed that overexpression of FKBP5 caused a significant increase in mRNA (Figure 4B) and protein expression (Figure 4C, 4D) of C/EBPα and PPARγ (p<0.05), while the results of the inhibition of FKBP5 expression were the opposite of the above. These results verify that FKBP5 has a regulatory effect on the differentiation of Yanbian yellow cattle preadipocytes.

### FKBP5 targeted by bta-miR-365-3p participates in the AMPK/mTOR signaling pathway in the regulation of preadipocyte differentiation in Yanbian yellow cattle

**Figure 5:**
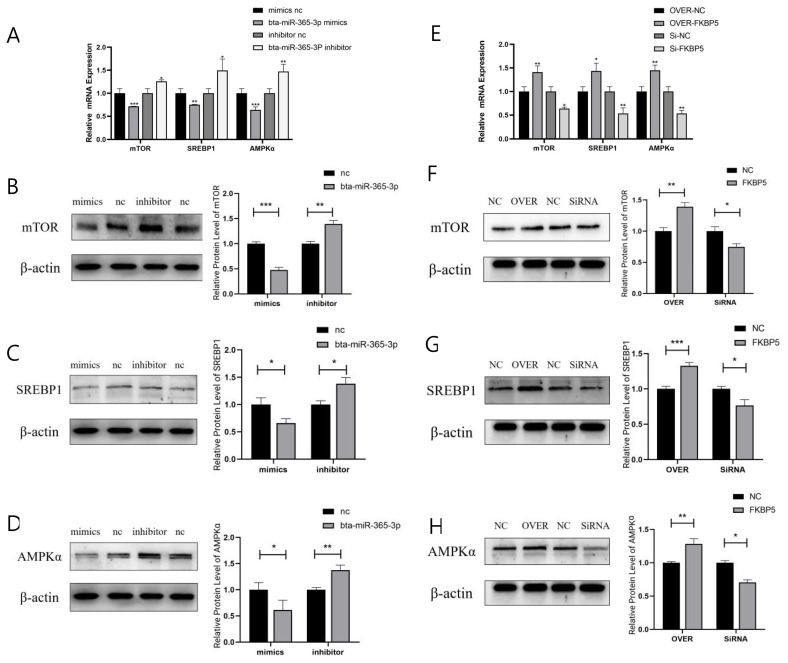
bta-miR-365-3p targeting of FKBP5 participates in the AMPK/mTOR signaling pathway in the regulation of preadipocyte differentiation in Yanbian yellow cattle. Effects of overexpression and inhibition of bta-miR-365-3p on AMPKα, mTOR, and SREBP1 mRNA expression. (B, C, D) Effects of overexpression and inhibition of bta-miR-365-3p on AMPKα, mTOR, and SREBP1 protein expression. (E) Effects of overexpression and inhibition of FKBP5 on AMPKα, mTOR, and SREBP1 mRNA expression. (F, G, H) Effects of overexpression and inhibition of FKBP5 on AMPKα, mTOR, and SREBP1 protein expression. * p<0.05; ** p<0.01; *** p<0.001.

RNA and protein were extracted on the fourth day of induction of differentiation. The expression levels of AMPKα, mTOR, and SREBP1 were detected by qRT-PCR and Western blot. The results showed that overexpression of bta-miR-365-3p significantly inhibited the expression of mRNA (Figure 5A) and protein (Figure 5B–5D) of AMPKα, mTOR, and SREBP1 (p<0.05), while the results of inhibition of bta-miR-365-3p expression were the opposite of the above results. Overexpression of FKBP5 significantly increased mRNA (Figure 5E) and protein expression (Figure 5F–5H) of AMPKα, mTOR, and SREBP1 (p<0.05), while inhibition of FKBP5 expression was contrary to the above results.

## DISCUSSION

Adipose is an important part of lipid storage, energy homeostasis, and systemic insulin sensitivity [19]. It is important to understand the differentiation and function of adipose. *In vitro* culture systems of preadipocytes can objectively elucidate the key conditions of adipocyte formation in vivo, and miRNA plays an irreplaceable role in this process.

Micro-RNAs are a kind of non-coding RNA with a length of about 23 nucleotides. As an important regulatory factor of adipogenesis and lipid metabolism, miRNAs can participate in the signaling pathways related to adipogenesis, regulate the development of adipocytes, thus promoting [20] or inhibiting [21] the differentiation of adipocytes. Li et al [22] found that miR-27a in sheep could inhibit *PPARγ*, *SCD*, *LPL*, *FABP4*, and other genes involved in lipid synthesis by targeting the *CPT1B* gene and significantly inhibit the formation of lipid droplets. Zhang et al [23] confirmed that miR-181a targeted the *TGFBR1* gene through TGFβ/Smad pathway to promote the differentiation of porcine preadipocytes. All the above studies show that miRNA plays an important role in the process of fat formation in livestock and poultry. The intramuscular fat content in steers of Yanbian yellow cattle is significantly higher than that of bulls [24], and our research group confirmed the differential expression of miR-365-3p in the longissimus dorsi muscles of bulls and steers. These results suggest that miR-365-3p may be related to fat deposition in cattle, so miR-365-3p was selected in this experiment to explore its role in lipid formation. The study showed that overexpression of bta-miR-365-3p could significantly down-regulate the expression of *PPARγ* and *C/EBPα* genes. In contrast, the effect of inhibiting the expression of bta-miR-365-3p was opposite to that of overexpression of bta-miR-365-3p. This indicated that bta-miR-365-3p may play an important role in the adipogenic differentiation of Yanbian yellow cattle. Oil red O staining experiment and triglyceride content detection experiments showed that the lipid droplets and triglyceride content of Yanbian yellow cattle preadipocytes were decreased after transfection with bta-miR-365-3p mimics, which indicated that bta-miR-365-3p mimics significantly inhibited the accumulation of lipid droplets in bovine preadipocytes. All the above results indicate that bta-miR-365-3p plays a negative regulatory role in the differentiation of preadipocytes in Yanbian yellow cattle.

FKBP5 plays an important role in immune regulation, the occurrence and treatment of cancer, and the regulation of steroid hormones [25]. It has been found that FKBP51 knockout could inhibit the differentiation and synthesis of adipocytes by regulating PPARγ activity and resist the fat accumulation in mice induced by high-fat diet [26]. In addition, compared with wild-type mice, the embryonic fibroblasts of FKBP51 knockout mice showed significant differentiation resistance, almost no lipid accumulation, and the expression of adipogenic genes was significantly reduced [27]. All the above studies show that FKBP5 plays an important role in adipocyte differentiation. In this experiment, the overexpression of FKBP5 significantly increased the mRNA and protein expression of C/EBPα and PPARγ, but the results of inhibiting FKBP5 expression were the opposite. The results show that FKBP5 may promote the differentiation of Yanbian yellow cattle adipocytes, which is consistent with the above research results.

Bioinformatics analysis showed that the 3′UTR region of FKBP5 had a targeted binding site with bta-miRNA-365-3p seed sequence, and some studies showed that FKBP5 was related to adipogenesis [26]. Therefore, it is speculated that bta-miRNA-365-3p may affect the differentiation process of Yanbian yellow cattle preadipocytes by targeting FKBP5. In this study, the direct targeting relationship between bta-miRNA-365-3p and FKBP5 was verified by double luciferase reporter gene detection. In addition, the results of qRT-PCR and Western blot analysis showed that overexpression of bta-miRNA-365-3p could inhibit the expression of FKBP5, while inhibition of bta-miRNA-365-3p could promote the expression of FKBP5. The results further verified that bta-miR-365-3p had a negative regulatory targeting relationship with *FKBP5* gene.

Genes like *PPARs*, *C/EBPs*, *SREBPs* are considered as the key regulatory factors in the early stage of lipogenesis, and AMPK/mTOR and Wnt/β-actin signaling pathways are considered as the key signaling pathways which can regulate lipogenesis [28]. Adenosine 5′-monophosphate-activated protein kinase (AMPK) is an important kinase in cells and a switch of energy metabolism, which is related to lipid regulation in the liver [29]. mTOR participates in the energy metabolism of animals by influencing the energy state of cells [30]. SREBPs are not only a transcription factor regulating the synthesis and utilization of fatty acids, triglycerides, and cholesterol, but also a key regulator of the mTOR pathway, which plays an important role in regulating the lipid metabolism of adipocytes. It could be seen that AMPK/mTOR signaling pathway has an important influence on adipocyte differentiation, so the AMPK/mTOR signaling pathway was selected to verify that bta-miR-365-3p participates in the regulation of adipocyte differentiation of Yanbian yellow cattle by targeting FKBP5. In the early stage of the experiment, the same amount of bta-miR-365-3p mimics, bta-miR-365-3p inhibitor, FKBP5 overexpression vector, FKBP5-siRNA and the control samples of the above experimental groups were transfected into Yanbian yellow cattle preadipocytes. RNA and protein from cells induced to differentiate up to day 4 were later extracted to detect changes in the expression of *AMPKα*, *mTOR*, *SREBP1*, and other signaling pathway node genes by qRT-PCR and Western blot. The results showed that the overexpression of bta-miR-365-3p significantly inhibited the mRNA and protein expression of *AMPKα*, *mTOR*, and *SREBP1* genes, but the inhibition of bta-miR-365-3p expression was contrary to the above results. Overexpression of FKBP5 significantly increased the expression of *AMPKα*, *mTOR* and *SREBP1* genes, while inhibition of FKBP5 expression was contrary to the above results. The results of the bta-miR-365-3p targeting relationship and regulation of FKBP5 shown above confirmed that bta-miR-365-3p negatively regulates FKBP5. To sum up, bta-miR-365-3p may be involved in AMPK/mTOR signaling pathway to regulate the differentiation of Yanbian yellow cattle preadipocytes by targeting the *FKBP5* gene.

## CONCLUSION

In a word, bta-miR-365-3p participates in AMPK/mTOR signaling pathway by directly targeting the 3′UTR region of FKBP5 and regulates the differentiation of preadipocytes in Yanbian yellow cattle. This study provides a reliable theoretical basis for the molecular mechanism of intramuscular fat deposition in cattle. Bta-miR-365-3p and FKBP5 may play a role as candidate genes in improving beef quality. However, the expression and interaction of genes, proteins and metabolites all have complex mechanisms. Next, we will further explore the interaction network between bta-miR-365-3p and its target gene, and further verify its mechanism through animal experiments.

## Figures and Tables

**Table 1:** SiRNA sequence primers of *FKBP5* gene

Designation	SiRNA sequence primers
Negative control	F: 5′-UUCUCCGAACGUGUCACGUTT-3′
	R: 5′-ACGUGACACGUUCGGAGAATT-3′
FKBP5-BOS-308	F: 5′-GGGAAAUUGUCAACUGGAATT-3′
	R: 5′-UUCCAGUUGACAAUUUCCCTT-3′
FKBP5-BOS-453	F: 5′-GCAAACCAGAAUAUGCAUATT-3′
	R: 5′-UAUGCAUAUUCUGGUUUGCTT-3′
FKBP5-BOS-819	F: 5′-GGAAGCCUAAAUUUGGCAUTT-3′
	R: 5′-AUGCCAAAUUUAGGCUUCCTT-3′

*FKBP5*, FK506-binding protein 5.

**Table 2:** Primers used for quantitative real-time polymerase chain reaction analysis

Gene designation	Genbank accession NO.	Primer sequence	Annealing temperatures (°C)	Product size (bp)
*FKBP5*	NM_001192862.1	F: 5′-CCGCTCGAGGCTGCTTTGTTCTGGTATG-3′	55	191
		R: 5′-CAGTCTAGAATTTCTTAGGCTGTGGGAC-3′		
*C/EBPα*	NM_176784.2	F: 5′-TGGACAAGAACAGCAACGAG-3′	60	127
		R: 5′-TCACTGGTCAACTCCAGCAC-3′		
*PPARγ*	NM_181024.2	F: 5′-CGAGAAGGAGAAGCTGTTGG-3′	60	122
		R: 5′-TCAGCGGGAAGGACTTTATG-3′		
*mTOR*	NM_001014909.1	F: 5′-TGAACTGGAGGCTGATGGACAC-3′	60	83
		R: 5′-TGACTGGCCAGCAGAGTAGGAA-3′		
*AMPKα*	XM_024981363.1	F: 5′-ACCATTCTTGGTTGCTGAAACTC-3′	60	80
		R: 5′-CACCTTGGTGTTTGGATTTCTG-3′		
*SREBP1*	NM_001113302	F: 5′-CTTGGAGCGAGCACTGAATTG-3′	60	83
		R: 5′-GGGCATCTGAGAACTCCTTGTC-3′		
*β-actin*	NM_173979.3	F: 5′-AGGCATCCTGACCCTCAAGTA-3′	60	95
		R: 5′-GCTCGTTGTAGAAGGTGTGGT-3′		

## Data Availability

The data are available by sending an email to the corresponding author.

## SUPPLEMENTARY MATERIAL

**Supplementary Table S1**. Primary antibodies used for Western Blot
