# Supplementary material for: Bta-miR-365-3p-targeted FK506-binding protein 5 participates in the AMPK/mTOR signaling pathway in the regulation of preadipocyte differentiation in cattle
Source: Anim Biosci. 2024 Apr 24;37(7):1156–67. doi: 10.5713/ab.23.0328 (PMC11222839; doi:10.5713/ab.23.0328)
Supplement: Supplementary file 1 [file ab-23-0328-Supplementary-Table-1.pdf]

Supplementary Table 1. Primary antibodies used for Western Blot

Primary antibodies	Company	Dilution ratio	Species	Catalog Number
Rb a SREBP1	Bioss	1:500-1:2000	Cattle	bs-1402R
AMPK-alpha Rabbit mAb	Cell Signaling Technology	1:500-1:2000	Cattle	5832
CRBPA Rabbit pAb	ABdonal	1:500-1:2000	Cattle	A0904
Rb a Phspho-mTOR	Bioss	1:500-1:2000	Cattle	bs-1992R
PPAR gamma Ab	Affinity	1:500-1:2000	Cattle	bs-0530R
FKBP5 Rabbit mAb	Cell Signaling Technology	1:1000	Cattle	12210
β-Actin (4D3) monoclonal antibody	Bioworld	1:5000-1:20000	Cattle	BS6007M
